# Intrinsic biotic factors and microsite conditions drive seedling survival in a species with masting reproduction

**DOI:** 10.1002/ece3.5861

**Published:** 2019-12-04

**Authors:** Francesco Martini, Chaobo Zou, Uromi Manage Goodale

**Affiliations:** ^1^ Guangxi Key Laboratory of Forest Ecology and Conservation College of Forestry Guangxi University Nanning China; ^2^ State Key Laboratory of Conservation and Utilization of Subtropical Agro‐bioresources College of Forestry Guangxi University Nanning China

**Keywords:** aspect, initial seedling height, maple, Mast seeding, plant population and community dynamics, seedling mortality, soil seed bank, subtropical forest, survival analysis

## Abstract

1. Seedling recruitment following a masting event, where more fruits are produced in synchrony and intermittently compared with other species, plays a crucial role in determining species diversity and community structure. Such seedling recruitment can be superabundant, but followed by high mortality shortly thereafter. Differences in biotic factors such as seedling characteristics, competition, and herbivory, and microsite‐specific abiotic factors could determine seedling fate in space and time.
2. In a subtropical forest in south China, for 2 years using censuses conducted every 1–2 months, we monitored 40 seed traps and 120, 1 m^2^ quadrats in five 1‐ha plots located from 1,400 to 1,850 m asl for the masting maple species, *Acer campbellii* subsp. *sinense* (Pax) P.C.DeJong. We measured biotic—conspecific and heterospecific seedling density, species richness, herbivory, seedling height, and leaf number—and abiotic—canopy openness, slope, and aspect—factors to assess drivers of seedling survival and evaluated *A. campbellii* subsp. *sinense* presence in the soil seed bank (SSB).
3. The masting seed dispersal peak and seedling emergence peak occurred between October 2017 and January 2018, and May 2018, respectively. Of 688 selected seedlings, mortality was 92.7% within one year. No seeds were observed in the SSB. Seedling height and leaf number positively affected seedling survival, while seed placement as measured by aspect also showed effects on survival. Conspecific and heterospecific density and herbivory did not show any clear effect. Higher probabilities of seedling survival were found in areas with larger canopy openness (≥12% canopy gap size) and in steeper microsites (≥35°).
4. *Synthesis*. Masting is mainly studied as a population‐level phenomenon from the fruiting tree perspective. Our study of individual seedling fate revealed that intrinsic biotic factors and seed placement were key drivers of survival. Although biotic determinants such as competition from conspecifics or heterospecifics or herbivory did not determine survival, their ubiquitous presence may be an underlying equalizer in community dynamics where seedlings that overcome biotic pressures, if placed at the right microsite, are at better odds at being recruited to the next life history stages.

## Graphical abstract

We monitored seed rain, soil seed bank, and seedling recruitment and survival for the masting maple species, *Acer campbellii* subsp., in a subtropical forest in South China. The masting seed dispersal peak and seedling emergence peak occurred between October 2017 and January 2018, and May 2018, respectively. Seedling mortality was 92.7% within 1 year, and we found no seeds in the SSB. Our study of individual seedling fate revealed that intrinsic biotic factors—seedling height and leaf number—and seed placement—aspect—were key drivers of survival.

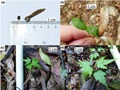

## 1 INTRODUCTION

The seedling stage represents a crucial bottleneck in plant establishment, where most individuals do not survive to the next ontogenetic stage (Harms, Wright, Calderón, Hernández, & Herre, 2000; Queenborough, Burslem, Garwood, & Valencia, 2007). In some species, seedlings can be superabundant following an interannual and synchronous reproductive episode of high seed production (masting) (Kelly & Sork, 2002). This evolutionary strategy influences forest regeneration and affects animal populations (Curran & Leighton, 2000; Zwolak, Witczuk, Bogdziewicz, Rychlik, & Pagacz, 2018) with cascading effect on species diversity and composition. The recruitment potential of a species is not only characterized by its ability to succeed in seed production, but also in seed persistence in the soil seed bank (SSB) after reaching the forest floor and the seedlings’ ability to survive and wait as advanced regeneration (Clark et al., 1999). The SSBs represent biodiversity reservoirs allowing seed dispersal not only in space, but also in time, and temporally uncouple seed fate from masting following recruitment events (Vandvik, Klanderud, Meineri, Måren, & Töpper, 2016). Masting has been especially investigated from the seed or fruit production, seed dispersal, and seed survival perspectives focusing on its evolutionary significance (Kelly & Sork, 2002; Pearse, Koenig, & Kelly, 2016). Studies that investigated the drivers of seedling survival following a masting event coupled with the assessment of advanced regeneration and persistence in the SSB are limited (Cleavitt, Battles, Fahey, & Blum, 2014; Oshima, Tokumoto, & Nakagawa, 2015).

While it is hypothesized that masting could provide an evolutionary advantage through predator satiation and dispersal efficiency (Kelly & Sork, 2002), high seedling mortality is usually recorded shortly after germination, limiting recruitment (Abrams & Johnson, 2013; Green & Newbery, 2002). For both masting and nonmasting species, factors that determine recruitment can be complex, species‐specific, and change along both temporal and spatial axes (Fricke, Tewksbury, & Rogers, 2014; Oshima et al., 2015; Zhu et al., 2018). Biotic factors that influence seedling survival can be intrinsic depending on the seed's resources, seedling vigor, or extrinsic such as conspecific or heterospecific competition and seed predation or seedling herbivory (Comita et al., 2014; Downey, Lewis, Bonsall, Fernandez, & Gripenberg, 2018; Khan, 2004; Lu et al., 2015). Larger seeded species are known to have a greater advantage in survival over smaller seeded species (Moles & Westoby, 2004). In a masting cohort, larger seeds that can give rise to larger seedlings could provide an advantage in securing growing space over comparatively smaller members. Host‐specific herbivores and pathogens can act in a density‐ and/or distance‐dependent manner to reduce seedlings and juvenile plant survival, when seedlings are found close to conspecific adult trees or in areas of high conspecific density (conspecific negative density dependence—CNDD, Janzen, 1970; Connell, 1971). This hypothesis has received support in some studies (Johnson et al., 2014; Terborgh, 2012) but has found mixed support in seedling populations following a mast seedling episode (Norghauer & Newbery, 2016; Oshima et al., 2015).

Abiotic conditions can be as equally important as biotic elements in securing recruitment success. Light—often measured as canopy openness—is one of the most important abiotic factors that affects seedling survival (Beckage & Clark, 2003; Lu, Wang, Yu, Zhang, & Zhu, 2018). In masting species, the mosaic distribution of light in the forest floor due to canopy gaps may create a clumped distribution of surviving seedlings. For example, it has been shown that larger canopy gaps favor seedling survival in the first year after germination in masting in dipterocarp species in Borneo (Oshima et al., 2015) and a beech species in North America (Cleavitt, Fairbairn, & Fahey, 2008), indicating that the first year following a masting event is a crucial time in the recruitment of the new cohort.

Other microsite conditions, such as topographical features of the location where seeds fall, can also influence germination and seedling recruitment success in space and time by modulating what resource are available and the duration of resources availability. For example, Frey, Ashton, McKenna, Ellum, and Finkral (2007) found that slope position had clear effects on tree seedling survival in five different species in North America. Ma et al. (2014) also reported a significant slope effect on tree survival. Yet, other studies that assessed seedling survival did not detect any effect of slope on mortality risk (Lin, Comita, Johnson, Chen, & Wu, 2017). Another topographical site characteristic, aspect, has been shown to influence plant association (Badano, Cavieres, Molina‐Montenegro, & Quiroz, 2005; Warren, 2008), but not adult tree survival (Wang et al., 2012) or mortality (Wu, Franklin, Liu, & Lu, 2017). Recruitment following a masting event is commonly assessed as a percentage of seedlings that survive at a given point in time (for example, Frey et al., 2007; Green & Newbery, 2002). Studies that assess several predictors—both abiotic and biotic—to explain what is driving survival of a masting species along a temporal axis are rare (but see Cleavitt et al., 2014; Norghauer & Newbery, 2016; Oshima et al., 2015).

Our objective was to investigate the regeneration process, through the assessment of seed rain, SSB, and seedlings, of a species with masting behavior and to determine the drivers of regeneration. We used *Acer campbellii* subsp. *sinense* (Pax) P.C.DeJong, an endemic maple species in a subtropical forest in south China, as our model system. We evaluated the importance of the masting event in comparison with seeds that may be persistent in the SSB and advanced regeneration present on the forest floor as a remnant seedling cohort from previous regeneration events. We monitored seed rain and seedling recruitment for 2 years that included a masting event in 2017. We then followed seedling survival of the newly germinated cohort over a 1‐year period to assess which biotic or abiotic factors influence seedling population dynamics. We tested the following hypotheses:

H 1 After a masting event, once seeds have germinated, intrinsic biotic factors, for example, seedling height and number of developed leaves, act as key predictors of survival success and may be more important than extrinsic biotic factors, such as conspecific and heterospecific competition or herbivory.

H 2 Abiotic factors in the microsite position where seeds fall and germinate, measured as microhabitat topographical features of slope and aspect and canopy openness, can further determine their recruitment success.

## 2 MATERIALS AND METHODS

### 2.1 Study area

**Figure 1:**
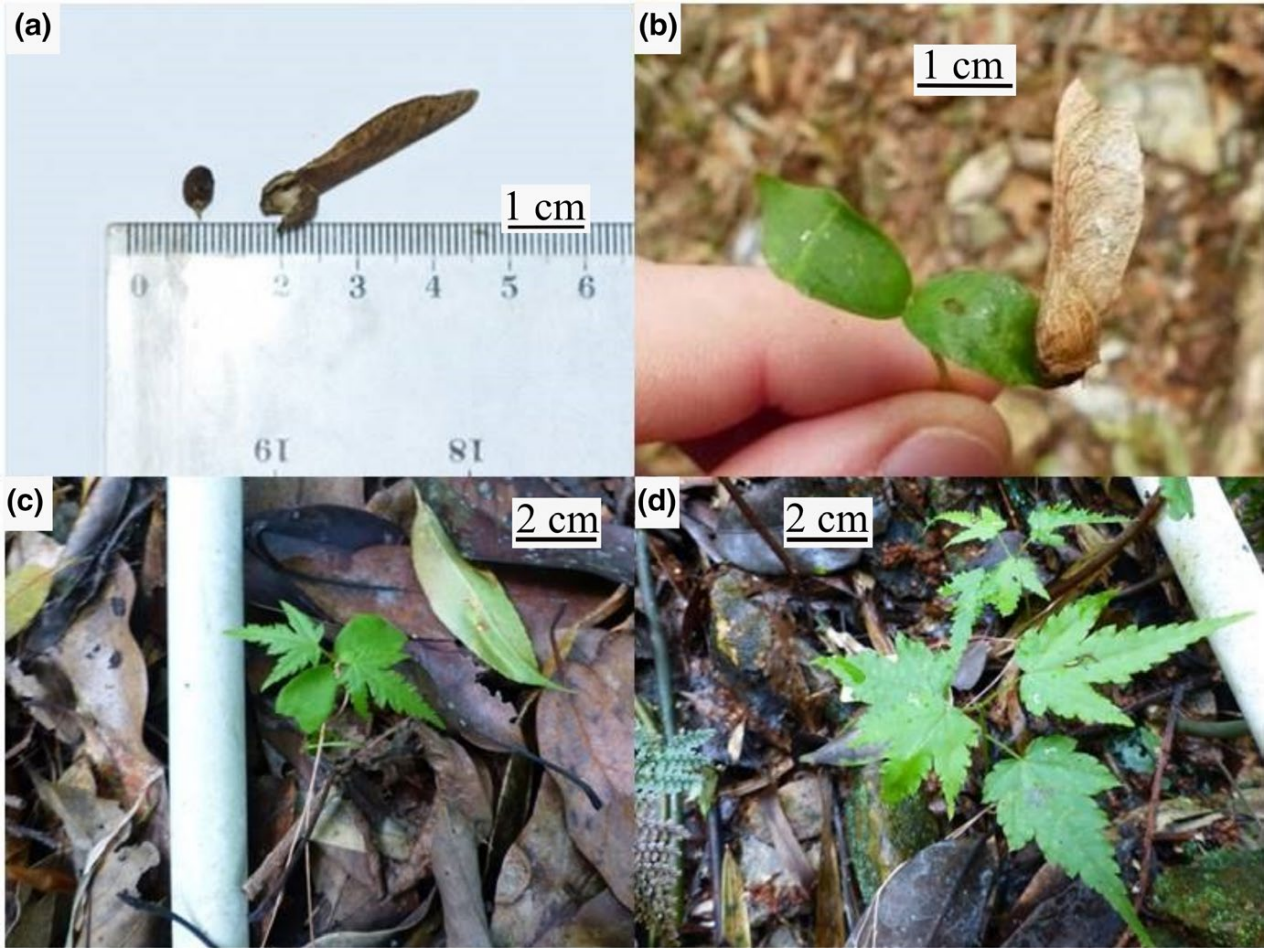
Study species, *A. campbellii* subsp. *sinense* (a) samara and seed, (b) newly germinated seedling with samara still attached, (c) seedling with two cotyledons, and (d) seedlings with fully developed leaves

This study was conducted in the subtropical forest of Cenwanglaoshan National Nature Reserve (24°21′ – 24°32′N, 106°15′ – 106°27′E), Guangxi Zhuang Autonomous Region, in south China. The reserve spans across a mountainous area of almost 300 km^2^, with elevation ranging from 400 to 2,062 m asl. The average annual rainfall ranges from 1,190 mm at lower elevations to 1,857 mm above 1,500 m asl. Average annual temperature varies from 20°C at low elevations to 14°C at high elevations. During our seedling survival assessment period (May 2018 – May 2019), mean temperature (T) was 16.4°C (−2.5 to 30.7°C), and total rainfall was 2,207 mm (HOBO U30 Weather Station, Onset Computer Corporation). The vegetation presents differences with increasing elevation, ranging from subtropical deciduous broad‐leaved forest at low elevations to subtropical evergreen forest in the midelevations and deciduous broad‐leaf mixed forests at the high elevations. However, most low elevation forest have been commercially exploited (BirdLife International, 2009). This study was conducted at elevations from 1,400 m up to 1,850 m asl, in the deciduous broad‐leaf mixed forests. *A. campbellii* subsp. *sinense* (Figure 1) is found at elevations between 500 and 2,500 m asl in the deciduous broad‐leaf mixed forests (eFloras, 2008).

### 2.2 Data collection

Our experimental design was implemented taking advantage of a series of 1‐ha permanent monitoring plots that have been recently established as part of a long‐term monitoring project on forest regeneration mechanisms in Guangxi Province. The 5‐ha plots studied here had 20,649 individuals above 1 cm dbh, belonging to 310 tree species out of which 36 individuals were *A. campbellii* subsp. *sinense*, with higher abundance in the lowest plot. We constructed 40 seed traps and 120 seedling quadrats, in five 1‐ha plots found at 1,400, 1,500, 1,550, 1,750, and 1,850 m asl, following the standardized design employed by the Center for Tropical Forest Science seed rain and regeneration monitoring stations (Harms et al., 2000). Each seed trap, built using PVC frames and using a nylon net of 1 mm mesh size, and measuring 0.5 m^2^, was positioned approximately 0.7 m from the forest floor, and three seedling quadrats, measuring 1 m^2^, were set up secured to the forest floor, in three perpendicular directions and 2 m far from the seed trap (the seed trap and three seedling quadrat are referred to as census station). At each elevation, eight census stations were placed at the corner and in the middle of each side of the 1‐ha plot and 2 m away from the plot perimeter. Seed traps and seedling quadrats were monitored for a total of 18 censuses starting from May 2017 until May 2019, at 1‐ to 2‐month census intervals. At each census, seed traps were emptied into labeled bags and transferred to the laboratory of Guangxi University. There, *A. campbellii* subsp. *sinense* samaras were counted and separated into samaras with or without a full seed inside. In each seedling quadrat, all woody seedlings (shrubs and trees) of <50 cm height from soil surface were tagged and measured. Each seedling was scored as alive if it contained green leaves or buds and was identified using a tag containing a unique identification number, and seedling height was measured as the distance from soil surface to the top of the plant. At each census, height and leaf number assessments were continued and each new recruit was added to the census data scored as “1,” with dead individuals scored as “0.” Following species or morpho‐species identification, species’ seedling counts were used to calculate conspecific density, heterospecific density, and species richness at the 1‐m^2^ level.

We calculated topographical data as slope and aspect at the seedling quadrat level using a clinometer and a compass, respectively. Above each seedling quadrat, we measured canopy openness using a spherical densitometer (Lemmon, 1956) two times, in January and August 2018.

In August 2018, at the fourth census, once the majority of seed germination from the masting event was completed, we measured herbivory damage by insects. This allowed us to test the effect of herbivores in already established individuals which survived the initial critical bottleneck. Leaf damage was visually estimated for all seedlings leaves following six, a priori defined percentage classes (0%, <5%, <25%, <50%, <75%, ≥75%; Schuldt et al., 2015), accounting for a wide variety of damage types from different insect feeding guilds. This assessment may underestimate damage types that do not remove significant amounts of photosynthetic tissue (e.g., from leaf sap‐sucking insects such as aphids). Mean herbivory values per individual were calculated and used in the leaf level assessments. The herbivory assessment was conducted by a single observer (F. Martini), to exclude observer bias, and visual images were used to correlate observer values with actual percentage damage during the initial training period. In total, 466 leaves from 157 individuals were measured. A summary of explanatory variables is presented in Table 1.

**Table 1:** Predictor variables used in the generalized linear mixed model with mean values ± standard error (*SE*) per 1‐ha plot. The data presented are calculated for the 1‐m^2^ seedling quadrats that included the studied species only. Therefore, values come from 20 seedling quadrats in the 1‐ha plot at 1,550 m, 21 for the plot at 1,750 m, and 20 for the 1‐ha plot at 1,850 m. For canopy openness and slope, presented in the survival analysis, we show the mean values ± *SE* of each category by 1‐ha plot. The seedlings sample size is presented in parenthesis for the seedling traits

	1,550 m	1,750 m	1,850 m
Canopy openness (%)	14.53 ± 0.61	12.95 ± 0.50	13.84 ± 0.59
Small	10.34 ± 0.84	10.60 ± 0.46	10.85 ± 0.25
Large	14.99 ± 0.58	14.12 ± 0.0.45	15.45 ± 0.46
Slope (°)	33.91 ± 1.82	27.13 ± 1.66	24.24 ± 2.52
Flat	NA	9	4.27 ± 1.47
Medium	15.2	16.6	14.47 ± 2.18
Steep	29.5 ± 0.94	27.16 ± 1.03	29 ± 1.30
Ridge	40.89 ± 1.64	41.20 ± 3.60	36.53 ± 0.37
Northness (*N* = 1)	0.04 ± 0.16	−0.05 ± 0.18	0.32 ± 0.16
Eastness (*E* = 1)	−0.40 ± 0.13	−0.29 ± 0.11	−0.28 ± 0.16
Seedling conspecific density (*n*/m^2^)	10.80 ± 2.31	6.71 ± 1.14	17.80 ± 3.6
Seedling heterospecific density (*n*/m^2^)	13.5 ± 6.49	9.33 ± 4.59	2.85 ± 0.58
Species richness (*n*/m^2^)	1.85 ± 0.21	3.10 ± 0.34	1.75 ± 0.32
Seedling height (cm)	6.74 ± 0.11 (212)	5.75 ± 0.12 (128)	5.98 ± 0.09 (348)
Seedling leaf (*n*)	3.01 ± 0.09 (212)	3.48 ± 0.10 (128)	3.34 ± 0.06 (348)
Herbivory (%)	17.7 ± 2.28 (48)	14.41 ± 1.49 (40)	23.61 ± 2.17 (76)

For the analysis of persistence of seeds in the SSB following the masting event, we collected soil samples in October 2017, May 2018, and November 2018 from the four corners surrounding each seedling quadrat. Soil cores were obtained using a 10‐cm depth soil probe with a 5 cm diameter. Each sample was individually tagged and collected in sealed plastic bag and transported to Guangxi University where they were sieved with a 2‐mm mesh sieve to remove stones, twigs, or roots and then spread in 10 × 15 × 5 cm plastic trays filled with sterilized potting soil and gross sand (Ter Heerdt, Verweij, Bekker, & Bakker, 1996). The samples were allowed to germinate in greenhouse shelves and watered regularly. Seedling emergence was scored for 5 months at weekly intervals for the first month and biweekly for the following 4 months. Then, the soil samples were dried for a few days, remixed, and watered and assessed for germination for another month (Ter Heerdt et al., 1996). At the end, the soil was searched again for any remaining seeds to complete germination assessment, before samples were discarded.

### 2.3 Statistical analyses

All statistical analyses were conducted in R (version 3.6.1, R Core T, 2019). We used generalized linear mixed models (GLMM) with a binomial distribution and a log‐link function to assess the relative importance of biotic versus abiotic factors on seedling survival using the *lme4* R package (Bates et al., 2019). Seedling survival was modeled as a binary response variable (1 = survived, 0 = dead) using initial seedling height, initial leaf number, conspecific density, heterospecific density, species richness, canopy openness, slope, and aspect as predictors. All predictors were used as continuous variables in the GLMM. Aspect was transformed to eastness and northness following Schwarz, Fahey, and McCulloch (2003), with sin(aspect), bounded between 1 for due east to –1 for due west, as a measure of eastness, and cos(aspect), bounded between 1 for due north to –1 for due south, as a measure of northness. All predictors were centered and scaled prior to data analysis. We used data only from the seedlings germinated in May 2018 following the masting event, and we further restricted the analysis on three of the five one‐hectare plots (1,550, 1,750, and 1,850 m asl, which contained 688 seedlings from 61 quadrats) because the two lowest plots presented only six (at 1,400 m) and four (at 1,500 m) *A. campbellii* subsp. *sinense* individuals, respectively. We used Moran's I test to assess the presence of spatial autocorrelation, and given the low *p*‐value, we accounted for it using a seedling quadrat as random factor in the models. We first used a random intercept model with seedling quadrat nested into census station, following Chen et al. (2010). However, to remove singularity and convergence issues we finally kept only seedling quadrat as a random factor, which also provided a better model fit. We did not include 1‐ha plot as a random factor as we only had three “blocks” in the model, and it has been suggested a minimum of five levels to accurately estimate the variance among blocks (Harrison, 2015). The *p*‐values of the Moran's I test after the addition of the random factor were higher indicating that the model now correctly accounted for the hierarchical experimental design and assured spatial independence. We modeled survival for the full year period (Model A). Then, because herbivory was measured once seedlings were established, we included herbivory as an explanatory variable together with all other factors for modeling seedling survival using census data from the fourth census in August 2018, when herbivory was measured, to the last measured census in May 2019 (Model B).

For both models, we followed a model selection approach using the Akaike's information criterion (AIC) to retain the best‐fit model. To confirm our selection and to test the effects of our explanatory variables, we used the model averaging method (Burnham & Anderson, 2002) with the R package *MuMIn* (Barton, 2019). The global model, with all explanatory variables and all possible combinations of variables were set as candidates for model selection. Coefficients that had 95% confidence intervals that do not include zero were considered as representing strong relationships. We retained the more conservative model within ΔAIC values <2 that included variables having >0.8 model‐averaged importance of terms. However, models within two AIC units of the minimum may also be considered to show strong support.

Seedling survival probability was analyzed using the R package *survival* (Therneau, 2019), using the nonparametric Kaplan–Meier method, for the full year period. For this analysis, we transformed the assessed environmental predictors—canopy openness and slope—into categorical variables. We divided slope as flat (<10°), medium (10–19°), steep (20–35°), and ridge (≥35°), and we classified canopy openness into two groups as small gaps (<12%) and larger gaps (≥12%). We decided on this threshold because we considered it to be a meaningful separation between more close and open gaps, and because it allowed us to have a more balanced sample size for each gap class.

Figures were produced through the packages *ggplot2* (Wickham, 2009) and *survminer* (Kassambara & Kosinski, 2018).

## 3 RESULTS

**Figure 2:**
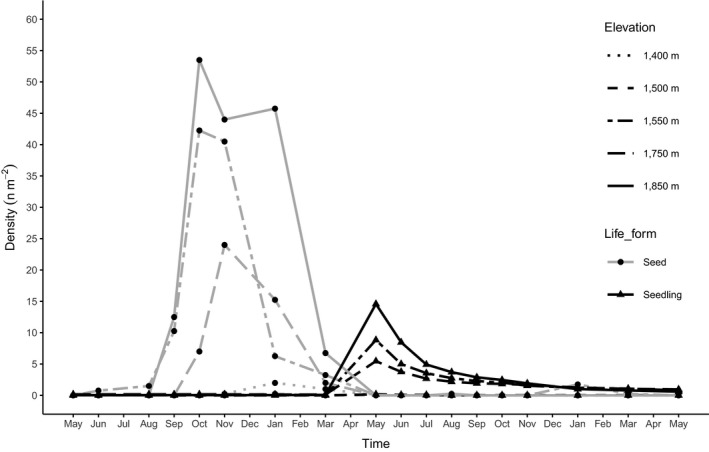
Seed rain and seedling density (n/m^2^) recorded at each census during the study period (May 2017 – May 2019). Seed line shows the *A. campbellii* subsp. *sinense* seeds (“developed”) collected at each field visit, while the seedling line represents all individual seedlings from all five, 1‐ha plots. Therefore, the seedlings in 1 month can include the same individuals in another month. Points represent censused months. Results are shown for each of the 1‐ha plots

During the monitoring period, we found 5,820 seeds from 97 morpho‐species that emerged in the SSB assessment, but we did not find any seeds of *A. campbellii* subsp. *sinense*. The number of seedlings detected in the seedling quadrats prior to the masting event remained very low with only six individual seedlings recorded in all 120 quadrats during the first year prior to seedling emergence from masting. Five of the six survived until the end of the experiment. During the mast seeding, 31 out of 40 seed traps contained a total of 1,612 *A. campbellii* subsp. *sinense* seeds (mean 9.54, range 1–74, per seed trap per sampling time; 79.65% of the samaras contained a fully developed seed, while 20.35% were classified as undeveloped), with the highest number of seeds collected in October and November 2017 (Figure 2). The peak in seed dispersal was followed by a unimodal seedling emergence peak in May 2018 with a total of 698 *A. campbellii* subsp. *sinense* individual seedlings recorded as new recruits following the masting event (Figure 2).

**Figure 3:**
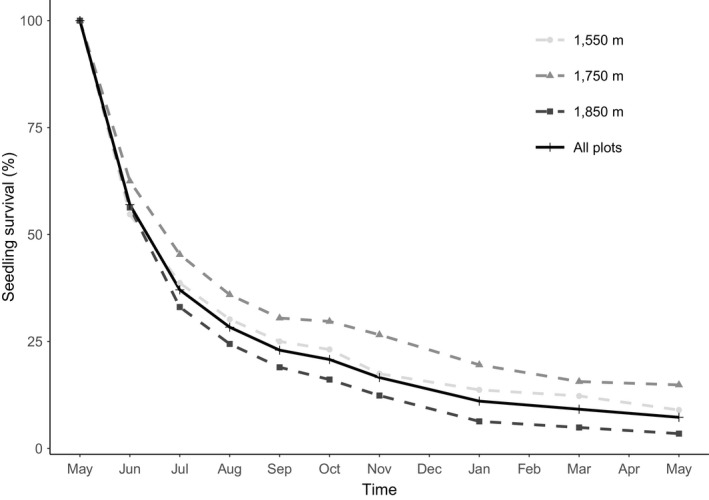
Seedling survival of *A. campbellii* subsp. *sinense* seedlings from May 2018 to May 2019 in Cenwanglaoshan Nature Reserve, for the three one‐hectare plots separately and combined. Points represent censused months

After June 2018, only four new seedlings were recorded, one of them during the last census (May 2019). For the analyzed seedling cohort, mortality was higher in the first 6 months with 83.4% of the newly recruited seedlings not surviving, and at the end of the first year, mortality of seedlings increased to 92.7% (Figure 3). The highest mortality occurred during the first month after the peak germination (43%, May–June 2018).

The most parsimonious models that we selected using the model selection were consistent to the models selected using the model averaging method (model‐averaged results are reported in Tables S1–S4). We found evidence to support H1 in our full year analysis, in that the intrinsic biotic factors were more strongly associated with seedling survival compared with the extrinsic biotic factors evaluated here (AIC = 311.2; Tables 2 and 3; model A). Seedling height and number of leaves were included in the final model for the first year (*n* = 688), while neither conspecific density nor heterospecific density or species richness were supported in the final model (Tables 2 and 3, model A). Initial seedling height was the most important factor, with taller individuals having higher survival (β = 0.64 ± 0.19, *p* < .001). The number of leaves also affected survival positively (β = 0.51 ± 0.19, *p* < .01). Among abiotic predictors, only cos(aspect), which represents northness, was moderately supported and presented a negative slope in the best model containing it (H2; β = −0.54 ± 0.31, *p* = .08).

**Table 2:** Model selection approach for generalized linear mixed model with binomial distribution for seedling survival. Response variable: seedling survival (Surv). Predictors: heterospecific density (HD), seedling initial height (Height*_i_*), seedling height at the time herbivory was measured (Height*_h_*), species richness (S), conspecific density (CD), canopy openness (CO), slope (Sl), aspect (Asp), seedling initial leaf number (L*_i_*), seedling leaf number at the time herbivory was measured (L*_h_*), and herbivory (Herb). Seedling quadrat was used as random factor. All predictors were centered and scaled before analysis. The most parsimonious model within a ΔAIC <2 selected is presented in bold font

Models evaluated for selection	AIC
Model (A) Full year model selection (*N* = 688)	
Surv ~ HD + Height*_i_* + S + CD + CO + Sl + sin(Asp) + cos(Asp) + L*_i_*	316.3
Surv ~ HD + Height*_i_* + S + CD + Sl + sin(Asp) + cos(Asp) + L*_i_*	314.5
Surv ~ HD + Height*_i_* + S + CD + Sl + cos(Asp) + L*_i_*	312.9
Surv ~ Height*_i_* + S + CD + Sl + cos(Asp) + L*_i_*	311.6
Surv ~ Height*_i_* + S + Sl + cos(Asp) + L***_i_***	311.4
Surv ~ Height*_i_* + Sl + cos(Asp) + L*_i_*	310.8
Surv ~ Height*_i_* + cos(Asp) + L*_i_*	310.1
**Surv ~ Height*_i_* + L*_i_***	**311.2**
Surv ~ Height*_i_*	316.9
Model (B) With herbivory: August 2018 – May 2019 (*N* = 157)	
Surv ~ HD + Height*_h_* + S + CD + CO +Sl + cos(Asp) + sin(Asp) + L*_h_* + Herb	183.4
Surv ~ Height*_h_* + S + CD + CO + Sl + cos(Asp) + sin(Asp) + L*_h_* + Herb	181.4
Surv ~ Height*_h_* + S + CD + CO + Sl + cos(Asp) + sin(Asp) + Herb	179.6
Surv ~ Height*_h_* + S + CD + CO + Sl + cos(Asp) + sin(Asp)	178.0
Surv ~ Height*_h_* + S + CD + CO + Sl + cos(Asp)	176.5
Surv ~ Height*_h_* + S + CD + Sl + cos(Asp)	175.0
Surv ~ Height*_h_* + S + Sl + cos(Asp)	175.3
Surv ~ Height*_h_* + Sl + cos(Asp)	174.1
**Surv ~ Height*_h_* + cos(Asp)**	**174.6**
Surv ~ Height*_h_*	177.3

**Table 3:** Coefficients of the best‐fit generalized linear mixed model explaining seedling survival from (A) the full analyzed year, from May 2018 to May 2019 and (B) August 2018 to May 2019, including herbivory. Response variable: seedling survival (Surv). Predictors: seedling initial height (Height*_i_*), seedling height at the time herbivory was measured (Height*_h_*), aspect (Asp), and seedling initial leaf number (L*_i_*)

Time interval	Model	Estimate	*SE*	*z*‐value	*p*
Model (A) Full year	Surv ~ Height*_i_* + L*_i_*				
	Intercept	−3.7922	0.5051	−7.507	<.001
	Height*_i_*	0.6373	0.1950	3.268	**.001**
	L*_i_*	0.5086	0.1863	2.731	**.006**
Model (B) Herbivory	Surv ~ Height*_h_* + cos(Asp)				
	Intercept	−1.4970	0.4263	−3.512	<.001
	Height*_h_*	0.7262	0.2607	2.786	**.005**
	cos(Asp)	−0.7335	0.3640	−2.015	**.04**

In our model including herbivory as an explanatory variable with all other measured variables, seedling height at the time herbivory was measured and cos(aspect) had the strongest support to determine seedling survival (AIC = 174.6; Tables 2 and 3; model B). Height and aspect effects (β = 0.73 ± 0.26, *p* = .005 and β = −0.73 ± 0.36, *p* = .04, respectively) were similar to the full year model results. In addition, slope showed some modest positive influence in the best model containing it (β = 0.50 ± 0.32, *p* = .12), meaning higher survival in steeper sites.

**Figure 4:**
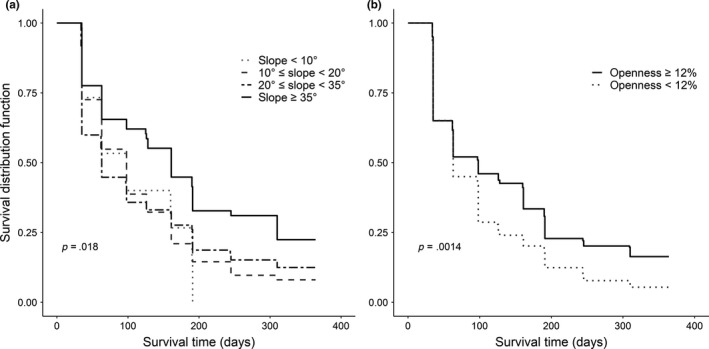
Seedling survival probabilities for *A. campbellii* subsp. *sinense* seedlings in different conditions of (a) slope and (b) canopy openness. The survival functions were calculated using the nonparametric Kaplan–Meier method. Note that the graph lines end when the survival probability does not change for a future census event

Although slope and canopy openness did not get selected in the best‐fit model for the full year analysis, our survival analysis using the Kaplan–Maier method for the full year data showed clear patterns. Survival probability differed across slope values (*p* = .018; Figure 4a), with increasing probability of survival in steeper slopes. Seedlings emerging in more open areas, as measured by canopy openness, had greater survival probability compared with smaller gaps (*p* = .0014; Figure 4b).

## 4 DISCUSSION

Our study provides comprehensive information on the regeneration ecology of a masting species, assessing advanced regeneration from previous recruitment events, the persistence in the SSB and seedling survival success following a mast seeding event. Most of the seeds fell in a time span of two months, and almost no seeds were produced the following year, indicating an asynchronous reproduction for *A. campbellii* subsp. *sinense*. Given that we did not find any seeds in the SSB before or after masting event, this could indicate that this species does not have the potential to persist in time (Saatkamp, Poschlod, & Venable, 2014), therefore having limited possibilities of regeneration outside of the limited masting time and subsequent seedling emergence. Although SSB has been evaluated in several studies, very rarely has it been specifically investigated in species with masting reproduction (but see Du, Guo, Gao, & Ma, 2007). Only 7.3% of the seedlings that emerged after the masting event survived after 1 year, a much lower number compared to other studies that reported on seedling survival following a masting event. For example, survival of *A. saccharum* has been reported to be as high as 87% in one study (Gardescu, 2003), while it was 30% in another (Cleavitt et al., 2014). In one of the few investigations on masting across temporal scales conducted in Asia, Oshima et al. (2015) observed seedling survival of 12 dipterocarp species, 1.5 years after mast fruiting in Borneo, to be between 12% and 60%. Similar to other reported studies (Frey et al., 2007; Oshima et al., 2015), we also found that seedling mortality was higher during the first few months following masting. This high mortality may be also driven by variables that were not measured in our study. For instance, fungi or other pathogens are known to play a significant role in seedling mortality (Fricke et al., 2014), but this was not assessed in this study. We recorded some damage on the seedling leaves that was not caused by insects (personal observation), but we did not measure it and was minor compared to damage from insect herbivory. Leaf damage by insects, even though it was not retained in the best‐fit models, was high (19.68% ± 1.31 *SE*) compared to other studies. For example, Schuldt et al. (2015) recorded a maximum of 17.8% damage in the most attacked trees of their species pool. Alternatively, the high mortality, which was likely consistent also with the previous masting event (only six individuals were found at the beginning of the experiment), might be due to an inherent maternal effect (Roach & Wulff, 1987), with adult trees consistently producing abundant, yet “weak,” seeds.

Our results supported H1, because intrinsic biotic factors, that is, seedling initial height and leaf number, explained seedling survival while the extrinsic factors, competition and predator pressure, did not find support when added to our global model. Initial height has been reported in previous studies as an important variable influencing seedling survival, both in species that undergo mast seeding/fruiting (Cleavitt et al., 2014; Oshima et al., 2015) and in species with other regeneration strategies (Comita et al., 2009; Lin et al., 2017; Pu, Zhu, & Jin, 2017). Initial number of leaves was an important factor in our best supported models for the full year period. Notably, while many studies reported seedling height as a relevant driver of seedling survival (Cleavitt et al., 2014; Lin et al., 2017), the effect of leaf number has been rarely tested on seedling survival. In our study, the two variables were not correlated (*r* = .08), which favors the idea of considering these factors separately. Because of the synchronous germination and the variability found in seed developmental stage of the seed rain assessment observed in our study, we assume that initial height and leaf production might be mainly explained by seed characteristics such as seed mass (Khan, 2004) or seed maturity. Indeed, seedling initial height and leaf number are considered as proxies of seedling vigor, derived from seed size and nutrition (Baraloto, Forget, & Goldberg, 2005). Yet, we lack experimental data to support this hypothesis, and we hope that future studies may further investigate this relationship.

Among biotic factors assessed in our study, neither conspecific density nor heterospecific density significantly affected masting seedling survival. The idea that greater conspecific density will result in greater mortality has received wide support in several ecosystems around the globe (Comita et al., 2014), and for some masting species (Oshima et al., 2015), though not in others (Gardescu, 2003; Norghauer & Newbery, 2016; Oshima et al., 2015). This may indicate that conspecific density only has a weak effect on determining seedling recruitment of a masting species. Similarly, heterospecific seedling density has also been reported to play an important role in tree seedling survival in some species (Bai et al., 2012), but its effect was not found to be ubiquitous (Johnson, Condit, Hubbell, Comita, & Johnson, 2017) and was not supported in our study.

Leaf damage by herbivores and pathogens has been described as a key driver of seedling survival in nonmasting species (Moles & Westoby, 2004; Terborgh, 2012). In our study, insect herbivory, measured on seedlings that had already survived the most significant regeneration bottleneck, did not show an effect on seedling survival. Nonetheless, we cannot exclude it as an important driver during the first months after germination because in every seedling we measured we found herbivore damage from at least one type of insect feeding guild.

The second most important factor having a relevant role on survival, aspect, was retained in both best‐fit models, confirming our hypothesis on the importance of local abiotic predictors in determining seedling survival (H2). Compared to other abiotic variables, aspect has not received much attention. Nonetheless, it has been shown to influence plant association (Badano et al., 2005; Warren, 2008), though not adult tree mortality (Wu et al., 2017) or survival (Wang et al., 2012). A weak effect of aspect was also detected affecting seedling survival in the masting sugar maple by Cleavitt et al. (2014), but the effect was stronger in our case. Slope and canopy openness were not retained in the full year model, while slope was included in the model including herbivory. Slope has been shown to affect the survival of five masting species in North America (Frey et al., 2007) but did not have clear effects on seedling survival in karst forest species in Taiwan (Lin et al., 2017). Light, here measured as canopy openness, was not included in our best‐fit models, contrary to our expectation as well as several other studies of seedling survival (Goodale, Berlyn, Gregoire, Tennakoon, & Ashton, 2014; Kobe & Vriesendorp, 2011), including in seedlings germinating after mast seeding episodes (Cleavitt et al., 2008).

Even though slope and canopy openness were not supported as predictors of seedling survival, further investigation of different levels of these two categorical variables provided some interesting insights. Survival probability was higher in steeper seedling quadrats compared with flatter ones. This result may indicate that *A. campbellii* subsp. *sinense* grows preferentially in ridges and steep areas. This effect is similar to what was found at the community level in a deciduous subtropical forest in China (Jin, Russo, & Yu, 2018), but differ from what was described for two maple species—*A. saccharum* Marsh. and *A. rubrum* L.—in Frey et al. (2007), which presented higher survival in flatter locations. There were also clear differences between smaller and larger canopy gaps, with higher probability of survival in areas with more open canopies (Lu et al., 2018). This result gives some indication that canopy openness actually plays an important role in the survival, but it is not as influential as other factors. Still, because we did not target specific locations based on openness, additional studies with a stronger focus on this variable may provide a more in depth understanding of how light availability may affect a masting species’ survival.

## 5 CONCLUSION

Our study demonstrates that intrinsic biotic factors play a greater role in determining the fate of seedlings in a masting species compared with extrinsic biotic factors or abiotic factors. Given that this species seeds were not seen to persist in the soil seed bank and only a few number of seedlings across the landscape as advanced regeneration, and because it registered high seedling mortality after 1 year, the masting event and the factors that operate on seedling survival have a profound effect on the species’ distribution.

## CONFLICT OF INTEREST

None declared.

## AUTHOR CONTRIBUTIONS

FM and UMG designed the study. FM and CZ collected the data. FM and UMG performed data analyses. FM wrote the first draft of the manuscript, and all authors contributed to revisions.

## Data Availability

Data used for the analyses of this study are available in Dryad Digital Repository: https://doi.org/10.5061/dryad.r7sqv9s7n.
