# Supplementary material for: Intrinsic biotic factors and microsite conditions drive seedling survival in a species with masting reproduction
Source: Ecol Evol. 2019 Dec 4;9(24):14261–72. doi: 10.1002/ece3.5861 (PMC6953690; doi:10.1002/ece3.5861)
Supplement: Supplementary file 1 [file ECE3-9-14261-s001.docx]

**Intrinsic biotic factors and micro-site conditions drive seedling survival in a species with masting reproduction**

Francesco Martini^1,2^, Chaobo Zou^1,2^, Uromi Manage Goodale^1,2^*

^1^ Guangxi Key Laboratory of Forest Ecology and Conservation, College of Forestry, Guangxi University, Daxuedonglu 100, Nanning, Guangxi 530004, China

^2^ State Key Laboratory of Conservation and Utilization of Subtropical Agro-bioresources, College of Forestry, Guangxi University, Daxuedonglu 100, Nanning, Guangxi 530004, China

Email addresses: FM: franmart12@hotmail.it; CZ: cb.zou@outlook.com; UMG: : uromigoodale@gxu.edu.cn

* Author for correspondence: UMG: uromigoodale@gxu.edu.cn; uromi.goodale@aya.yale.edu

**Electronic Supplementary Material**

**Table S1**. Best models within a ΔAIC < 2 from model averaging, their AIC, and their weight, which indicates the probability of the specific model being the best among those in the list. Response variable: Seedling survival (Surv). Predictors: heterospecific density (HD), seedling initial height (Height*_i_*) species richness (S), conspecific density (CD), slope (Sl), canopy openness (CO), aspect (Asp), seedling initial leaf number (L*_i_*).

Global model:

glmer(Surv ~ HD + Height*_i_* + S + CD + CO + Sl +sin(Asp) + cos(Asp)+ L*_i_* + (1|seedling quadrat), family = binomial)

| Model fixed factors | df | logLink | AIC | delta | weight |
| --- | --- | --- | --- | --- | --- |
| cos(Asp) + Height*_i_* + L*_i_* | 5 | -150.07 | 310.15 | 0 | 0.15 |
| cos(Asp) + Height*_i_* + L*_i_* + Sl | 6 | -149.41 | 310.82 | 0.67 | 0.11 |
| Height*_i_* + L*_i_* | 4 | -149.55 | 311.10 | 0.95 | 0.09 |
| cos(Asp) + Height*_i_* + L*_i_* + S | 6 | -151.58 | 311.16 | 1.01 | 0.09 |
| Height*_i_* + L*_i_* + S | 5 | -150.70 | 311.40 | 1.25 | 0.08 |
| cos(Asp) + Height*_i_* + L*_i_* + S + Sl | 7 | -148.72 | 311.44 | 1.29 | 0.08 |
| CD + cos(Asp) + Height*_i_* + L*_i_* | 6 | -147.79 | 311.58 | 1.44 | 0.07 |
| CD +cos(Asp) + Height*_i_* + L*_i_* + S + Sl | 8 | -149.80 | 311.61 | 1.46 | 0.07 |
| CD + cos (Asp) + Height*_i_* + L*_i_* + Sl | 7 | -148.85 | 311.69 | 1.55 | 0.07 |
| cos (Asp) + Height*_i_* + L*_i_* + HD | 6 | -149.95 | 311.91 | 1.76 | 0.06 |
| cos (Asp) + Height*_i_* + L*_i_* + sin(Asp) | 6 | -150.00 | 312.01 | 1.86 | 0.06 |
| Height*_i_* + L*_i_* + S + Sl | 6 | -150.02 | 312.04 | 1.89 | 0.06 |

**Table S2**. Importance of terms for the model averaging results for the full year period. Coefficients values are the full subset of models. Importance broadly represents the percentage of models each factor was included in for all models within a ΔAIC < 2. Acronym explanation is available in Table S1.

| Variable | Estimate | Std. Error | *P* | Importance |
| --- | --- | --- | --- | --- |
| Intercept | -3.7812 | 0.4842 | <0.001 |  |
| cos(Asp) | -0.4235 | 0.3637 | 0.024 | 0.77 |
| Height*_i_* | 0.6504 | 0.1960 | <0.001 | 1 |
| L*_i_* | 0.4918 | 0.1860 | 0.008 | 1 |
| Sl | 0.1632 | 0.2923 | 0.577 | 0.39 |
| S | 0.1620 | 0.2902 | 0.577 | 0.39 |
| CD | 0.0911 | 0.2687 | 0.735 | 0.22 |
| HD | -0.0089 | 0.0816 | 0.912 | 0.06 |
| sin(Asp) | 0.0075 | 0.0870 | 0.931 | 0.06 |

**Table S3**. Best models within a ΔAIC < 2from model averaging from August 2018 to May 2019, including herbivory. Here, we show the model AICs and their weight, which broadly indicates the probability of the specific model being the best model among those in the list. Response variable: Seedling survival (Surv). Predictors: heterospecific density (HD), seedling height at the time herbivory was measured (Height*_h_*), species richness (S), conspecific density (CD), canopy openness (CO), slope (Sl), aspect (Asp), seedling leaf number at the time herbivory was measured (L*_h_*), and herbivory (Herb).

Global model:

glmer(Surv ~ HD + Height*_h_* + S + CD + CO + Sl +sin(Asp) + cos(Asp)+ L*_h_* + Herb + (1|seedling quadrat), family = binomial)

| Model fixed factors | df | logLink | AIC | delta | weight |
| --- | --- | --- | --- | --- | --- |
| cos(Asp) + Height*_h_* + Sl | 5 | -82.06 | 174.13 | 0 | 0.19 |
| cos(Asp) + Height*_h_* | 4 | -83.30 | 174.60 | 0.48 | 0.15 |
| cos(Asp) + Height*_h_* + CD + S + Sl | 7 | -80.52 | 175.05 | 0.92 | 0.12 |
| cos(Asp) + Height*_h_* + S + Sl | 6 | -81.63 | 175.26 | 1.14 | 0.11 |
| cos(Asp) + Height*_h_* + CD + Sl | 6 | -81.65 | 175.30 | 1.17 | 0.11 |
| cos(Asp) + Height*_h_* + Herb + Sl | 6 | -81.93 | 175.87 | 1.74 | 0.08 |
| cos(Asp) + Height*_h_* + L*_h_* + Sl | 6 | -81.95 | 175.90 | 1.77 | 0.08 |
| cos(Asp) + Height*_h_* + S | 5 | -82.99 | 175.97 | 1.84 | 0.08 |
| cos(Asp) + Height*_h_* + Herb | 5 | -83.06 | 176.12 | 2.00 | 0.07 |

**Table S4**. Importance of terms for the model averaging results for the period between August 2018 and May 2019, which includes herbivory as a fixed factor. Coefficients values are the full subset of models. Importance broadly represents the percentage of models each factor was included in for all models within a ΔAIC < 2. Acronym explanation is available in Table S3.

| Variable | Estimate | Std. Error | *P* | Importance |
| --- | --- | --- | --- | --- |
| Intercept | -1.4953 | 0.4150 | <0.001 |  |
| cos(Asp) | -0.7434 | 0.3612 | 0.04 | 1 |
| H*_h_* | 0.7223 | 0.2618 | 0.006 | 1 |
| Sl | 0.3924 | 0.3940 | 0.577 | 0.70 |
| S | 0.1293 | 0.2919 | 0.66 | 0.31 |
| CD | 0.1097 | 0.2905 | 0.71 | 0.23 |
| Herb | -0.0230 | 0.1137 | 0.84 | 0.15 |
| L*_h_* | 0.0090 | 0.0730 | 0.90 | 0.08 |
